# Patients with preoperative asymptomatic pyuria are not prone to develop febrile urinary tract infection after ureteroscopic lithotripsy

**DOI:** 10.1186/s12894-021-00919-z

**Published:** 2021-11-11

**Authors:** Kuan-Jung Lin, Eric Y. H. Huang, I-shen Huang, Yu-Hua Fan, Chih-Chieh Lin, Tzu-Ping Lin, Hsiao-Jen Chung, Shing-Hwa Lu, Junne-Yih Kuo, Howard Hung-Hao Wu, Yen-Hwa Chang, Alex T. L. Lin, William J. S. Huang

**Affiliations:** 1 Division of Urology, Department of Surgery, Taoyuan General Hospital, Ministry of Health and Welfare, Taoyuan, Taiwan; 2 Department of Urology, Taipei Veterans General Hospital, No. 201, Sec. 2, Shipai Rd., Taipei City, 11217 Taiwan, ROC; 3 Department of Urology, College of Medicine and Shu-Tien Urological Research Center, National Yang Ming Chiao Tung University, Taipei, Taiwan

**Keywords:** Pyuria, Ureteral stone, Ureterorenoscopic lithotripsy, Urinary tract infections

## Abstract

**Background:**

This study aimed to evaluate the association of asymptomatic pyuria before ureterorenoscopic lithotripsy (URSL) with postoperative febrile urinary tract infection (UTI).

**Methods:**

This observational case–control study identified the patients undergoing URSL for ureteral stones between May 2011 and October 2015. The included patients were classified into two groups: the asymptomatic pyuria group (6–50 white blood cells [WBCs]/high-power field [HPF]) and the non-pyuria group (≤ 5 WBCs/HPF). All data were collected by reviewing medical records. Postoperative outcomes were collected in terms of febrile UTI, emergency visits, and stone-free rate.

**Results:**

A total of 232 patients were included, 101 in the pyuria group, 131 in the non-pyuria group. Two (0.9%) patients developed febrile UTI after URSL and 12 (5.2%) patients visited emergency department for URSL-related symptoms. The overall stone-free rate was 90.9%. There was no significant difference between the pyuria and non-pyuria groups regarding febrile UTI, emergency visits, and stone-free rate. Multivariate analysis revealed that pyuria was neither significantly associated with postoperative febrile UTI (OR = 1.03, 95% CI = 0.06–18.10, *P* = 0.98), nor with emergency visits (OR = 0.48, 95% CI = 0.13–1.85, *P* = 0.29).

**Conclusions:**

Compared to the patients with sterile urine prior to URSL, those with asymptomatic pyuria were not prone to develop febrile UTI after URSL.

## Background

Ureterorenoscopic lithotripsy (URSL) is a common urological management for ureteral stones. Although URSL provides satisfactory stone-free outcomes, it is also associated with an overall complication rate of 9–25% [1]. Febrile urinary tract infection (UTI) is one of the complications which is far from negligible. Asymptomatic bacteriuria is a well-documented risk factor for febrile UTI following urological procedures [2]. Both the European Association of Urology and the Infectious Diseases Society of America recommend treating asymptomatic bacteriuria before endoscopic urologic procedures [3, 4].

However, many patients with ureteral stones may present with asymptomatic pyuria instead of bacteriuria. Although current guidelines suggest the use of prophylactic antibiotics to reduce the rate of clinical UTI following URSL [3, 5, 6], it lacks consensus for the management of patients with asymptomatic pyuria. Furthermore, we usually use pyuria as a surrogate for bacteriuria in real world practice. For patients with asymptomatic pyuria of less than 50 white blood cells [WBCs]/high-power field [HPF] in urinalysis, whether to eradicate pyuria prior to the operation is usually a dilemma to urologists. Therefore, this study aimed to evaluate the association of pre-URSL asymptomatic pyuria with postoperative febrile UTI. The results would provide further insight regarding the appropriate treatment of asymptomatic pyuria prior to endoscopic urologic procedures.

## Methods

### Study subjects

This observational case–control study was ethically approved by the Institutional Review Board of the study hospital (TPVGH-IRB 2014-11-004C). Between May 2011 and October 2015, patients who underwent URSL for ureteral stones were included. Patients were excluded for any one of the following: presence of symptoms of febrile UTI before URSL, renal staghorn stone, younger than 18 years, pregnancy, and preoperative severe pyuria (> 50 WBCs/HPF). According to preoperative midstream urine sediment which was collected one day before operation, the included patients were classified into two groups: the asymptomatic pyuria group (6–50 WBCs/HPF) and the non-pyuria group (≤ 5 WBCs/HPF). All patients received the same prophylactic antibiotics before URSL (a single dose of intravenous cefazolin 1000 mg within 30 min prior to URSL) and after URSL (oral cephalexin 500 mg every 6 h for three days).

### Data collection and variables

A retrospective review of medical records was performed for the collection of patient data, including demographics (age and sex), body mass index, medical history, stone characteristics (laterality, number, size, location, and composition), operation time, double pigtail stent insertion, and pre- and intraoperative urine culture. Postoperative outcomes were collected in terms of febrile UTI, emergency visits, and stone free rate. Febrile UTI was defined as a body temperature of > 38 °C with pyuria or bacteriuria within two weeks after URSL. Bacteriuria was defined as ≥ 100,000 colony-forming units of uropathogens per mL of cultured urine. Emergency visit was recorded if the patient had visited emergency department for operation-related symptoms within two weeks after URSL, for example, fever, gross hematuria, or low abdominal pain. The status of stone free and the position of double pigtail stent were examined by plain abdominal radiography of the kidney, ureter, and bladder at postoperative day one.

### Statistical analysis

Continuous variables were presented as mean (standard deviation) and categorical variables as count (percentage). Comparisons of continuous variables were performed using either Mann–Whitney U test or two-sample *t* test for depending on the distributional normality of the data. Either Fisher’s exact test or Pearson's chi-square test was used for comparing categorical variables. Logistic regression analysis was used to determine the association of preoperative pyuria with postoperative outcomes (febrile UTI and emergency visits). Demographics, known risk factors [7], and variables with *P* < 0.1 in univariate models were taken as covariates in the multivariate models. Results of regression analyses are presented as odds ratio (OR) with 95% confidence interval (CI). A *P* value of < 0.05 was considered statistically significant. All statistical analyses were performed using SPSS software Version 26 (IBM Corp., Armonk, NY, USA).

## Results

A total of 232 patients were included, with 101 in the pyuria group and 131 in the non-pyuria group. There was no significant difference between the pyuria and non-pyuria groups in terms of demographics, clinical data, and stone characteristics, except that the pyuria group had significantly greater mean stone size (9.0 vs. 7.5 mm, *P* < 0.01) and longer operation time (57.6 vs. 48.1 min, *P* < 0.01) than the non-pyuria group (Table 1). None of the included patients were institutionalized, immunocompromised, had renal transplants, or catheters in the urinary tract.

**Table 1:** Demographics, clinical characteristics, and postoperative outcomes

Variables	Pyuria	Non-pyuria	*P*
Number	101	131	
Age (year)^a^	57.2 (13.7)	55.2 (12.7)	0.24
*Sex*			0.14
Male	65 (64.4)	96 (73.3)	
Female	36 (35.6)	35 (26.7)	
Post-menopausal women	28 (80.0)	29 (80.6)	0.95
Body mass index (kg/m^2^)^a^	27.1 (5.8)	26.2 (4.2)	0.18
Diabetes	18 (17.8)	24 (18.3)	0.92
Hypertension	43 (42.6)	41 (31.3)	0.08
Chronic kidney disease	2 (2.0)	0 (0)	0.19
*Laterality*			0.08
Right	38 (37.6)	59 (45.0)	
Left	60 (59.4)	72 (55.0)	
Bilateral	3 (3.0)	0 (0)	
*Number of stones*			0.16
1	94 (93.1)	123 (93.9)	
2	7 (6.9)	4 (3.1)	
3	0 (0)	3 (2.3)	
4	0 (0)	1 (0.8)	
Mean size of stones (mm)^a^	9.0 (4.6)	7.5 (3.8)	< 0.01
Size of the largest stone (mm)^a^	9.1 (4.6)	7.6 (3.8)	< 0.01
*Location of the largest stone*			0.10
Renal pelvis	3 (3.0)	4 (3.1)	
Upper third ureter	54 (53.5)	48 (36.6)	
Middle third ureter	19 (18.8)	27 (20.6)	
Lower third ureter	16 (15.8)	35 (26.7)	
Ureterovesical junction	9 (8.9)	17 (13.0)	
Operation time (min)^a^	57.6 (23.4)	48.1 (23.0)	< 0.01
Postoperative double pigtail stent insertion	98 (97.0)	122 (93.1)	0.18
Postoperative stone free	93 (92.1)	118 (90.1)	0.60
Postoperative febrile urinary tract infection	1 (1.0)	1 (0.8)	0.68
Postoperative emergency visit	3 (3.0)	9 (6.9)	0.18

^a^Data were presented as count (percentage) or mean (standard deviation)

The most stone composition was calcium oxalate monohydrate (64.0%), followed by calcium oxalate monohydrate mixed with carbonate apatite (14.0%) and calcium oxalate dehydrate (10.0%). There was no significant difference in stone composition between the two groups (*P* = 0.30). The pyuria group had similar proportion of preoperative positive urine culture (26.1% vs. 19.1%, *P* = 0.38), preoperative bacteriuria (10.1% vs. 2.1%, *P* = 0.09), and intraoperative bacteriuria (2.6% vs, 1.2%, *P* = 0.50) to the non-pyuria group.

Regarding the postoperative outcomes, the overall stone-free rate was 90.9%. Only two (0.9%) patients experienced febrile UTI after URSL and 12 (5.2%) patients visited emergency department for URSL-related symptoms. There was no significant difference between the two groups in terms of stone-free rate, frequency of febrile UTI, and emergency visits (Table 1). The results of univariate and multivariate analysis for the association of preoperative asymptomatic pyuria with postoperative outcomes were presented in Table 2. The presence of preoperative pyuria was neither significantly associated with postoperative febrile UTI (OR = 1.03, 95% CI = 0.06–18.10, *P* = 0.98), nor with emergency visits (OR = 0.48, 95% CI = 0.13–1.85, *P* = 0.29).

**Table 2:** Logistic regression analysis for the association of preoperative pyuria with postoperative outcomes

	Odds ratio (95% confidence interval)	*P*
*Febrile urinary tract infection*		
Unadjusted	1.30 (0.08–21.04)	0.85
Adjusted^a^	1.03 (0.06–18.10)	0.98
*Emergency visit*		
Unadjusted	0.42 (0.11–1.57)	0.20
Adjusted^a^	0.48 (0.13–1.85)	0.29

^a^Covariates included age, sex, number of stones, diabetes, and positive preoperative urine culture

## Discussion

Currently, there is no clear consensus for the treatment of asymptomatic pyuria before endoscopic urologic procedures. In this observational study, prophylactic antibiotics with preoperative single-dose cefazolin and postoperative 3-day cephalexin was applied. The results revealed that preoperative asymptomatic pyuria before URSL was not significantly associated with postoperative febrile UTI. It suggested that prophylactic antibiotics might be sufficient for asymptomatic pyuria before URSL and the harmful consequences from overuse of antibiotics could be avoided, for example, *Clostridium difficile* infection, toxicity, and antimicrobial resistance of bacterial strains.

Risk factors of infectious complications after URSL have been evaluated in many studies [8]. However, only a few studies investigated the predictive role of preoperative asymptomatic pyuria in postoperative febrile UTI. Our results were comparable with some prior reports. The study by Morokuma et al. showed that preoperative pyuria was not a risk factor of postoperative febrile UTI in the patients undergoing URSL [9]. In a study consisting of a propensity-score matched cohort, the patients with postoperative fever after URSL had similar amount of preoperative urine leukocyte to those without postoperative fever [10]. Bai et al. also found that the proportion of preoperative pyuria did not differ between the patients complicated with and without urosepsis following URSL [11]. However, in the study by Mitsuzuka et al., the results of multivariate analysis revealed that preoperative pyuria was significantly predictive of postoperative febrile UTI (OR = 3.62, 95% CI = 1.26–8.11, *P* = 0.017) [12]. The controversial results could be explained by the heterogeneous definition of pyuria and varied incidence of postoperative febrile UTI. Among studies, pyuria was defined as a categorical variable of ≥ 5 WBCs/HPF [9], of ≥ 10 WBCs/HPF [11], of multiple levels [12], or as a continuous variable [10]. The incidence of postoperative febrile UTI also widely varied from 0.8 [11] to 18.3% [12]. These reasons may contribute to the conflict results regarding risk factors of febrile UTI after URSL, not to mention various covariates in each study. Therefore, further investigation are warranted to explore the potential role of preoperative asymptomatic pyuria in infectious complications after endoscopic urologic procedures.

There were several limitations in this study. The first came from the observational study design. Potential reporting bias and selection bias could not be avoided. Additionally, the incidence of febrile UTI was very low, resulting in a wide 95% CI for OR. The results in the present study should be interpreted with caution. Furthermore, this was a single-institutional study, which limited the external validity of the results. Some important variables were not collected, for example, the degree of pyuria, the preoperative status of stent, and condition of glycemic control. Finally, all patients in this study received the same first-line antibiotic prophylaxis preoperatively and postoperatively and all patients were considered as low-risk of infectious complications. It deserves more research to compare different regimens of antibiotics and to focus the patients with different risk profiles.

## Conclusions

In this observational case–control study, the results revealed that asymptomatic pyuria before URSL was not significantly associated with postoperative febrile UTI. It suggested that the patients with asymptomatic pyuria before URSL, compared with those with sterile urine, did not prone to develop clinically significant UTI. Intense antibiotics might not be necessary for asymptomatic pyuria before endoscopic urologic procedures and potential harms from overuse of antibiotics could be avoided.

## Data Availability

All data generated or analysed during this study are included in this published article.

## Abbreviations

CI: Confidence interval
HPF: High-power field
OR: Odds ratio
URSL: Ureterorenoscopic lithotripsy
UTI: Urinary tract infection
WBC: White blood cell
